# Draft genome sequence of *Rubidibacter lacunae* strain KORDI 51-2^T^, a cyanobacterium isolated from seawater of Chuuk lagoon

**DOI:** 10.4056/sigs.4398180

**Published:** 2013-10-16

**Authors:** Dong Han Choi, Jee-Youn Ryu, Kae-Kyoung Kwon, Jung-Hyun Lee, Changhoon Kim, Charity M. Lee, Jae Hoon Noh

**Affiliations:** 1 Marine Biotechnology Research Division, Korea Institute of Ocean Science and Technology, Republic of Korea; 2 Macrogen Inc., Gasan-dong, Geumcheon-gu, Republic of Korea; 3 Policy Research Section, Korea Institute of Ocean Science and Technology, Republic of Korea; 4 Marine Ecosystem Research Division, Korea Institute of Ocean Science and Technology, Republic of Korea

**Keywords:** *Cyanobacteria*, phosphonate utilization, photoautotrophy, *Rubidibacter lacunae*, seawater

## Abstract

A photoautotrophic cyanobacterium, *Rubidibacter lacunae* was reported in 2008 for the first time. The type strain, KORDI 51-2^T^, was isolated from seawater of Chuuk lagoon located in a tropical area. Although it belonged to a clade exclusively comprised of extremely halotolerant strains by phylogenetic analyses, *R. lacunae* is known to be incapable of growth at high salt concentration over 10%. Here we report the main features of the genome of *R. lacunae* strain KORDI 51-2^T^. The genome of *R. lacunae* contains a gene cluster for phosphonate utilization encoding three transporters, one regulator and eight C-P lyase subunits.

## Introduction

*Rubidibacter lacunae* type strain KORDI 51-2^T^ (=KCTC 40015^T^=UTEX L2944^T^) is a photoautotrophic cyanobacterium isolated from lagoon seawater of Chuuk, Micronesia [1]. At this time, the genus *Rubidibacter* is comprised of a single isolate. Further, only three environmental 16S rRNA gene sequences in the NCBI showed relatively high sequence similarity of ca. 96% to 16S rRNA gene of the strain. Thus, the genus seems either to be a numerically rare cyanobacterium or, to exploit specific environments such as microbial mats. Actually, the most similar sequences (accession no. of DQ861063 and DQ861117 in GenBank) to *Rubidibacter* were obtained in microbial mats of a coastal hypersaline pool. Nonetheless, the strain KORDI 51-2^T^ is a non-extreme halotolerant member in the *Halothece* cluster, exclusively composed of extremely halophilic/halotolerant bacteria. Considering this contrasting phenotypic trait, genomic information of KORDI 51-2^T^ could provide a good clue to understand genomic adaptation of cyanobacteria at extreme salt condition. Here we present a summary of the genomic features of *R. lacunae* strain KORDI 51-2^T^.

## Classification and features

**Figure 1:**
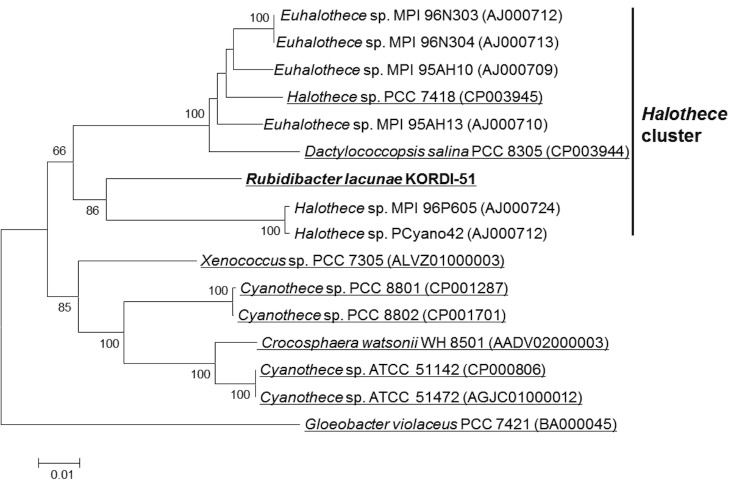
Neighbor-joining tree showing the phylogenetic position of *Rubidibacter lacunae* KORDI 51-2^T^ relative to other close cyanobacterial strains. GenBank accession numbers for each strain are shown in parenthesis. The tree uses the Jukes-Cantor corrected distance model to construct a distance matrix. Bootstrap values above 60%, based on 1,000 resamplings, are shown at the branching points. Strains with genome sequence are underlined.

**Figure 2:**
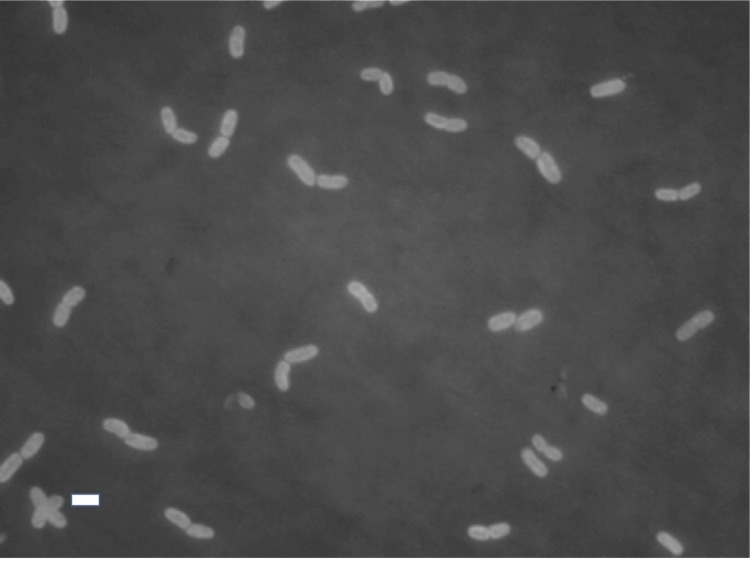
Epifluorescence micrograph of *R. lacunae* KORDI 51-2^T^. The picture was taken under green excitation and then converted to gray scale. Bar, 3 μm.

By phylogenetic analysis of 16S ribosomal RNA genes (Figure 1), *R. lacunae* KORDI 51-2^T^ was clustered into the *Halothece* cluster. Four *Euhalothece* strains belonging to the cluster were isolated from a hypersaline pond (strains MPI 96N303 and MPI 96N304) or a solar evaporation pond (strains MPI95AH10 and MPI95AH13) in Mexico [2]. These strains showed sustained growth between 6-16% salinity, and several strains could grow even in NaCl saturated brine, suggesting that they are at least extremely halotolerant cyanobacteria [2]. *Dactylococcopsis salina* and other *Halothece* strains belonging to the cluster were also isolated from various hypersaline environments, such as a solar lake in Egypt, a solar evaporation pond in Spain and hypersaline lagoon in Australia [2,3]. On the contrary, *R. lacunae* KORDI 51-2^T^ was isolated from natural seawater and able to grow at a salinity between 2 and 7% (Table 1). In addition, *R. lacunae* KORDI 51-2^T^ contains phycoerythrin, which differentiated it from the other strains belonging to the ‘*Halothece’* cluster [1]. The epifluorescence micrograph of the cells and other classification and general features were shown in Figure 2 and Table 1, respectively.

**Table 1:** Classification and general features of *R. lacunae* strain KORDI 51-2^T^ according to the MIGS recommendations [4]

**MIGS ID**	**Property**	**Term**	**Evidence code**^a^
		Domain *Bacteria*	TAS [5]
		Phylum *Cyanobacteria*	TAS [6-8]
		Class *Cyanobacteria*	TAS [8,9]
	Current classification	Order Unknown	
		Family 1.1	TAS [7]
		Genus *Rubidibacter*	TAS [1]
		Species *Rubidibacter lacunae*	TAS [1]
		Type strain KORDI 51-2	TAS [1]
	Gram stain	Not reported	
	Cell shape	Rods	TAS [1]
	Motility	None	TAS [1]
	Sporulation	None	IDA
	Temperature range	25-35^o^C	TAS [1]
	Optimum temperature	30^o^C	TAS [1]
MIGS-6	Habitat	Seawater	TAS [1]
MIGS-6.3	Salinity	2-7% (optimum: 5)	TAS [1]
MIGS-22	Oxygen Carbon source Energy source	Aerobic Autotroph Phototroph	TAS [1] TAS [1] TAS [1]
MIGS-15	Biotic relationship	Free living	TAS [1]
MIGS-14	Pathogenicity	None	NAS
MIGS-4	Geographic location	Chuuk state, Micronesia	TAS [1]
MIGS-5	Sample collection time	July, 2004	IDA
MIGS-4.1	Latitude	7^o^ 13’ N	IDA
MIGS-4.2	Longitude	151^o^ 58’ E	IDA
MIGS-4.3	Depth	40 m	IDA
MIGS-4.4	Altitude	Not applicable	NAS

a) Evidence codes - IDA: Inferred from Direct Assay; TAS: Traceable Author Statement (i.e., a direct report exists in the literature); NAS: Non-traceable Author Statement (i.e., not directly observed for the living, isolated sample, but based on a generally accepted property for the species, or anecdotal evidence). These evidence codes are from the Gene Ontology project [9].

## Genome sequencing and annotation

### Genome project history

The organism was selected for sequencing on the basis of its phylogenetic position. The genome project was deposited in the Genomes On Line Database [10] and draft genome sequence was deposited in GenBank database (accession number ASSJ00000000). The genome sequencing was carried out in Macrogen Inc. (Seoul, Korea) using GS-FLX Titanium sequencing technology. Table 2 presents the project information and its association with MIGS version 2.0 compliance [4].

**Table 2:** Genome sequencing project information

**MIGS ID**	**Property**	**Term**
MIGS-31	Finishing quality	Draft
MIGS-28	Libraries used	Shotgun library
MIGS-29	Sequencing platforms	454 GS-FLX Titanium
MIGS-31.2	Sequencing coverage	30×
MIGS-30	Assemblers	Newbler version 2.3
MIGS-32	Gene calling method	Prodigal, GenePRIMP
	Genbank ID	ASSJ00000000
	Genbank Date of Release	October 7, 2013
	GOLD ID	Gi22154
	Project relevance	Cyanobacterial ecology

### Growth conditions and DNA isolation

*R. lacunae* KORDI 51-2^T^ was grown in a 50 ml culture flask filled with 50 ml of modified f/2 medium in which silicate was omitted and ammonium chloride was supplemented (final conc. of 100 μM). The culture flask with inoculum was incubated at 25^o^C at about 20 μE m^-2^ s^-1^ (light:dark=14:10) for 3 weeks. Genomic DNA was isolated using Qiagen Genomic-tip 100/G (Qiagen) according to the manufacturer’s instruction.

### Genome sequencing and assembly

The genome was sequenced by pyrosequencing (GS-FLX Titanium). A shotgun library was constructed according to GS FLX Titanium Sequencing Method Manual. The 291,414 pyrosequencing reads obtained has an average length of 442.12 bp and were assembled using the Newbler assembler (version, 2.3; Roche) with default options. The final assembly resulted in 126 contigs longer than or equal to 500 bp with the contigs sum of 4,215,105 bp. After removing 27 short contigs with low coverage in order to minimize possible contamination, the remaining 99 contigs were used for further analyses (Table 3).

**Table 3:** Genome statistics

**Attribute**	**Value**	**% of total^a^**
Genome size (bp)	4,153,658	
DNA Coding region (bp)	3,323,928	80.02
DNA G+C content (bp)	2,335,216	56.22
No. of contigs	99	
Total genes^b^	3,790	
RNA genes	50	1.32
Protein-coding genes	3,740	98.68
Genes with functional prediction	2411	63.61
Genes with enzymes	775	20.45
Genes with transporter classification	343	9.05
Genes assigned to COGs	2,228	58.79
Genes assigned to Pfam	2,511	66.25
Genes assigned to TIGRFam	976	25.75
Genes assigned in paralog clusters	2427	64.04
Genes with signal peptides	137	3.61
Genes with transmembrane helices	810	21.37

a) The total is based on either the size of the genome in base pairs or the total number of protein coding genes in the annotated genome.

b) Also includes 283 pseudogenes.

### Genome annotation

The gene prediction and functional annotation of the genome sequence was basically performed within the Integrated Microbial Genomes – Expert Review (IMG-ER) platform [11]. The tRNAScan-SE was used to find tRNA genes [12]. Ribosomal RNA genes and ncRNA were predicted using RNAmmer [13] and Infernal [14] using the Rfam model [15], respectively. Identification of protein coding genes was performed using Prodigal [16], followed by a round of manual curation using the JGI GenePRIMP pipeline [17]. The predicted CDS were searched using the TIGR-fam, Pfam and COG databases implemented in the IMG systems.

## Genome properties

**Figure 3:**
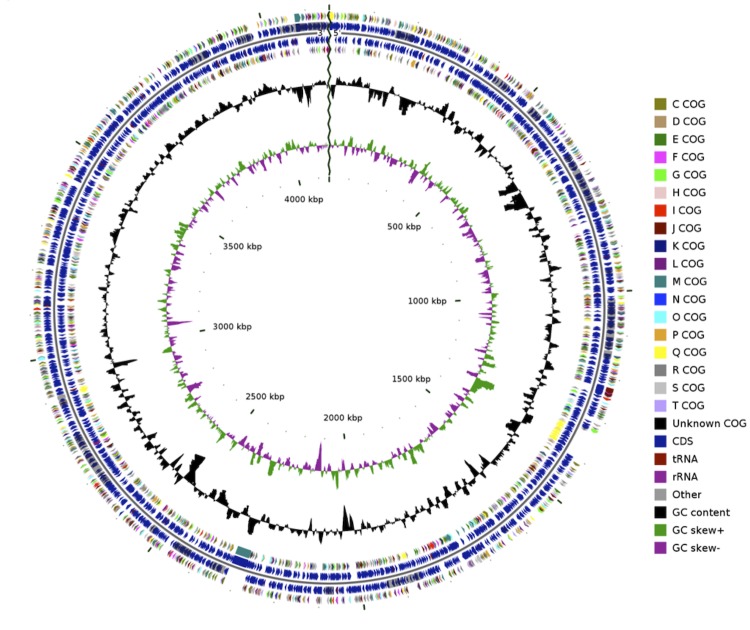
Graphical circular map of the genome. From outside to the center: color by COG categories and RNAs on forward strand, genes on forward strand, genes on reverse strand, color by COG categories and RNAs on reverse strand, GC content, GC skew.

The draft genome of *R. lacunae* KORDI 51-2^T^, with a total of 4.15 Mbp from 99 contigs, contains 56.22% G+C contents (Figure 3 and Table 3). A total of 3,790 genes were predicted. Of these, 283 pseudogenes. The remaining 3,457 were annotated as protein-coding genes and 50 for RNA genes (3 for rRNA, 41 for tRNA and 6 other nc RNA). The properties and the statistics of the genome are summarized in Table 3. The distribution of genes into COGs functional categories is presented in Table 4.

**Table 4:** Number of genes associated with the 25 general COG functional categories

**Code**	**Value**	**% age**	**Description**
J	149	6.08	Translation
A	1	0.04	RNA processing and modification
K	109	4.45	Transcription
L	127	5.18	Replication, recombination and repair
B	1	0.04	Chromatin structure and dynamics
D	24	0.98	Cell cycle control, mitosis and meiosis
Y	0	-	Nuclear structure
V	43	1.75	Defense mechanisms
T	105	4.28	Signal transduction mechanisms
M	166	6.77	Cell wall/membrane biogenesis
N	25	1.02	Cell motility
Z	1	0.04	Cytoskeleton
W	0	-	Extracellular structures
U	62	2.53	Intracellular trafficking and secretion
O	118	4.81	Posttranslational modification, protein turnover, chaperones
C	149	6.08	Energy production and conversion
G	126	5.14	Carbohydrate transport and metabolism
E	172	7.01	Amino acid transport and metabolism
F	58	2.37	Nucleotide transport and metabolism
H	157	6.4	Coenzyme transport and metabolism
I	51	2.08	Lipid transport and metabolism
P	158	6.44	Inorganic ion transport and metabolism
Q	75	3.06	Secondary metabolites biosynthesis, transport and catabolism
R	331	13.5	General function prediction only
S	244	9.95	Function unknown
-	1562	41.21	Not in COGs

## Insights from the genome sequence

**Figure 4:**
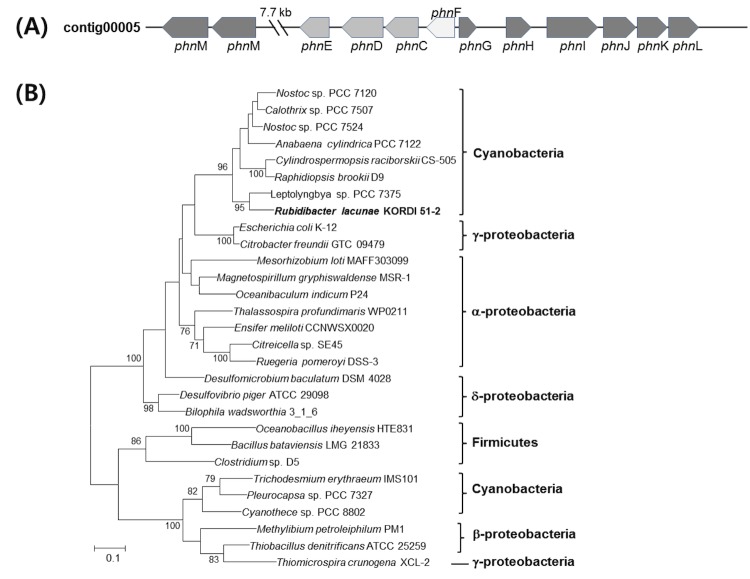
DNA topology of the *phn* cluster (A) and phylogenetic analysis of the PhnJ protein (B). **A**, Genes encoding phosphonate transport (gray), regulation (light gray), and the C-P lyase subunits (dark gray) are shown. Additional two sets of transporters were not shown. **B**, Phylogenetic relationship of the PhnJ protein from a variety of bacteria determined by maximum-likelihood analysis. Bootstrap values >70 are shown at the nodes. The scale bar represents amino-acid substitution per site.

A genome analysis of *R. lacunae* KORID 51-2^T^, revealed that it contains a gene cluster participating in organic phosphonate utilization. Likewise with a marine nitrogen-fixing cyanobacterium, *Trichodesmium erythraeum* IMS101 [18], the strain KORDI 51-2^T^ has orthologs to *phn*C-E (transporters) and *phn*G-M (C-P lyase complex) (Figure 4A). Additionally, an ortholog to *phn*F (transcriptional regulator) is found in strain KORDI 51-2^T^, but not in *T. erythraeum* IMS101. Phylogenetic analysis of PhnJ proteins found in various bacterial strains, showed that PhnJ proteins of cyanobacteria form polyphyletic lineages (Figure 4B), suggesting that the *phn* gene cluster of cyanobacteria might be acquired by horizontal gene transfer. As KORDI 51-2^T^ can grow in media supplemented with variety of organic phosphonate substrates (2-aminoethylphosphonate, methylphosphonate, phosphonoacetic acid and phosphonoformic acid) as a sole P-source (data not shown), the strain must be able to cleave C-P bonds of organic phosphonate by C-P lyase pathways and utilize them as a P-source.

## References

[1] Choi DH Noh JH Lee CM Rho S *Rubidibacter lacunae* gen. nov., sp. nov., a unicellular, phycoerythrin-containing cyanobacterium isolated from seawater of Chuuk lagoon, Micronesia. Int J Syst Evol Microbiol 2008; 58:2807-2811 10.1099/ijs.0.65798-0 19060063

[2] Garcia-Pichel F Nübel U Muyzer G The phylogeny of unicellar, extremely halotolerant cyanobacteria. Arch Microbiol 1998; 169:469-482 10.1007/s002030050599 9575232

[3] Allen MA Goh F Burns BP Neilan BA Bacterial, archaeal and eukaryotic diversity of smooth and pustular microbial mat communities in the hypersaline lagoon of Shark Bay. Geobiology 2009; 7:82-96 10.1111/j.1472-4669.2008.00187.x 19200148

[4] Field D Garrity G Gray T Morrison N Selengut J Sterk P Tatusova T Thomson N Allen MJ Angiuoli SV The minimum information about a genome sequence (MIGS) specification. Nat Biotechnol 2008; 26:541-547 10.1038/nbt1360 18464787 PMC2409278

[5] Woese CR Kandler O Wheelis ML Towards a natural system of organisms: proposal for the domains Archaea, Bacteria, and Eucarya. Proc Natl Acad Sci USA 1990; 87:4576-4579 10.1073/pnas.87.12.4576 2112744 PMC54159

[6] Castenholz RW. 2001. Oxygenic photosynthetic bacteria. In: Garrity GM, Boone DR, Castenholz RW (eds) Bergey’s Manual of Systematic Bacteriology 2nd ed. Vol 1, Springer-Verlag, New York, pp. 473-600.

[7] McNeill J, Barrie FR, Burdet HM, Demoulin V, Hawksworth DL, Marhold K, Nicolson DH, Prado J, Silva PC, Skog JE, *et al* International Code of Botanical Nomenclature, A.R.G. Ganter, Königstein, 2006, p. 1.

[8] Woese CR Stackebrandt E Macke TJ Fox GE A phylogenetic definition of the major eubacterial taxa. Syst Appl Microbiol 1985; 6:143-151 10.1016/S0723-2020(85)80047-3 11542017

[9] Ashburner M Ball CA Blake JA Botstein D Butler H Cherry JM Davis AP Dolinski K Dwight SS Eppig JT Gene ontology: tool for the unification of biology. The Gene Ontology Consortium. Nat Genet 2000; 25:25-29 10.1038/75556 10802651 PMC3037419

[10] Liolios K Chen IM Mavromatis K Tavernarakis N Hugenholtz P Markowitz VM Kyrpides NC The Genomes On Line Database (GOLD) in 2009: status of genomic and metagenomic projects and their associated metadata. Nucleic Acids Res 2010; 38:D346-D354 10.1093/nar/gkp848 19914934 PMC2808860

[11] Markowitz VM Mavromatis K Nvanova NN Chen IM Chu K Kyrpides NC IMG ER: a system for microbial genome annotation expert review and curation. Bioinformatics 2009; 25:2271-2278 10.1093/bioinformatics/btp393 19561336

[12] Lowe TM Eddy SR tRNAscan-SE: a program for improved detection of transfer RNA genes in genomic sequence. Nucleic Acids Res 1997; 25:955-964 9023104 10.1093/nar/25.5.955 PMC146525

[13] Lagesen K Hallin P Rødland EA Staerfeldt HH Rognes T Ussery DW RNAmmer: consistent and rapid annotation of ribosomal RNA genes. Nucleic Acids Res 2007; 35:3100-3108 10.1093/nar/gkm160 17452365 PMC1888812

[14] Nawrocki EP Kolbe DL Eddy SR Infernal 1.0: inference of RNA alignments. Bioinformatics 2009; 25:1335-1337 10.1093/bioinformatics/btp157 19307242 PMC2732312

[15] Griffiths-Jones S Moxon S Marshall M Khanna A Eddy SR Bateman A Rfam: annotating non-coding RNAs in complete genomes. Nucleic Acids Res 2005; 33:D121-D124 10.1093/nar/gki081 15608160 PMC540035

[16] Hyatt D Chen GL LoCascio P Land M Larimer F Hauser L Prodigal: prokaryotic gene recognition and translation initiation site identification. BMC Bioinformatics 2010; 11:119 10.1186/1471-2105-11-119 20211023 PMC2848648

[17] Pati A Ivanova NN Mikhailova N Ovchinnikova G Hooper SD Lykidis A Kyrpides NC GenePRIMP: a gene prediction improvement pipeline for prokaryotic genomes. Nat Methods 2010; 7:455-457 10.1038/nmeth.1457 20436475

[18] Dyhrman ST Chappell PD Haley ST Moffett JW Orchard ED Waterbury JB Webb EA Phosphonate utilization by the globally important marine diazotroph *Trichodesmium.* Nature 2006; 439:68-71 10.1038/nature04203 16397497

